# Integrative analysis of the ST6GALNAC family identifies GATA2-upregulated ST6GALNAC5 as an adverse prognostic biomarker promoting prostate cancer cell invasion

**DOI:** 10.1186/s12935-023-02983-x

**Published:** 2023-07-19

**Authors:** Meiqian Li, Zhihui Ma, Yuqing Zhang, Hanyi Feng, Yang Li, Weicong Sang, Rujian Zhu, Ruimin Huang, Jun Yan

**Affiliations:** 1 Model Animal Research Center, Medical School of Nanjing University, Nanjing University, Nanjing, China; 2 Center for Drug Safety Evaluation and Research, Shanghai Institute of Materia Medica, Chinese Academy of Sciences, Shanghai, China; 3 University of Chinese Academy of Sciences, Beijing, China; 4 Department of Urology, Shanghai Pudong Hospital, Fudan University Pudong Medical Center, Shanghai, China; 5 School of Chinese Materia Medica, Nanjing University of Chinese Medicine, Nanjing, China; 6 Department of Laboratory Animal Science, Fudan University, Shanghai, China; 7 Department of Urology, Shanghai Pudong Hospital, Fudan University Pudong Medical Center, Shanghai, China; 8 Model Animal Research Center, Nanjing University, Nanjing, China

**Keywords:** Prostate cancer, Metastasis, ST6GALNAC5, GATA2, Prognostic biomarker

## Abstract

**Background:**

ST6GALNAC family members function as sialyltransferases and have been implicated in cancer progression. However, their aberrant expression levels, prognostic values and specific roles in metastatic prostate cancer (PCa) remain largely unclear.

**Methods:**

Two independent public datasets (TCGA-PRAD and GSE21032), containing 648 PCa samples in total, were employed to comprehensively examine the mRNA expression changes of ST6GALNAC family members in PCa, as well as their associations with clinicopathological parameters and prognosis. The dysregulation of ST6GALNAC5 was further validated in a mouse PCa model and human PCa samples from our cohort (n = 64) by immunohistochemistry (IHC). Gene Set Enrichment Analysis, Gene Ontology, Kyoto Encyclopedia of Genes and Genomes and drug sensitivity analyses were performed to enrich the biological processes most related to ST6GALNAC5. Sulforhodamine B, transwell, luciferase reporter and chromatin immunoprecipitation (ChIP) assays were used to examine the PCa cell proliferation, invasion and transcriptional regulation, respectively.

**Results:**

Systematical investigation of six ST6GALNAC family members in public datasets revealed that ST6GALNAC5 was the only gene consistently and significantly upregulated in metastatic PCa, and ST6GALNAC5 overexpression was also positively associated with Gleason score and predicted poor prognosis in PCa patients. IHC results showed that (1) ST6GALNAC5 protein expression was increased in prostatic intraepithelial neoplasia and further elevated in PCa from a *PbCre;Pten*^*F/F*^ mouse model; (2) overexpressed ST6GALNAC5 protein was confirmed in human PCa samples comparing with benign prostatic hyperplasia samples from our cohort (p < 0.001); (3) ST6GALNAC5 overexpression was significantly correlated with perineural invasion of PCa. Moreover, we first found transcription factor GATA2 positively and directly regulated ST6GALNAC5 expression at transcriptional level. ST6GALNAC5 overexpression could partially reverse GATA2-depletion-induced inhibition of PCa cell invasion. The GATA2-ST6GALNAC5 signature exhibited better prediction on the poor prognosis in PCa patients than GATA2 or ST6GALNAC5 alone.

**Conclusions:**

Our results indicated that GATA2-upregulated ST6GALNAC5 might serve as an adverse prognostic biomarker promoting prostate cancer cell invasion.

**Supplementary Information:**

The online version contains supplementary material available at 10.1186/s12935-023-02983-x.

## Background

Prostate cancer (PCa) is one of the most common malignant cancers in men worldwide. In the United States alone, 288,300 new PCa cases were expected, and 34,700 men would die of this disease in 2023, showing a stepwise increase during past five years [1]. Localized PCa patients remain a 5-year relative survival rate > 99% after the standard treatments. Unfortunately, despite an initial favorable response, the majority of PCa patients who get treatment for metastatic PCa eventually develop resistance and progress [2]. The 5-year relative survival rate remains dismal for metastatic PCa patients at approximately 30%, and the clinical benefits are still unsatisfactory [1, 3]. Hence, there is an urgent need to explore novel diagnostic biomarker and/or therapeutic strategies for metastatic PCa.

Sialyltransferases are responsible for sialylation to transfer a sialic acid moiety from CMP-Neu5Ac to different acceptors, which is one of the most important glycosylations. The sialylation has been implicated in many biological processes, including cell–cell recognition and adhesion [4, 5]. Cumulative evidences have revealed critical roles of sialyltransferases in tumor development, tightly associated with multiple cancer hallmarks [6, 7]. For instance, our group reported that ST3GAL6 overexpression was negatively associated with luminal subtype of bladder cancer and required for cancer cells invasion [8], while ST3GAL5 overexpression was correlated with CD8^+^ T cell exhaustion in clear cell renal cell carcinoma [9]. Partially due to the dysregulated sialyltransferases in various cancers, some sialylated products have been reported to serve as cancer biomarkers. In PCa, the elevated levels of sialylated blood group antigen Sialyl Lewis X (SLe^X^) is linked with metastasis, while α-2,3-sialyl N-glycosylated prostate-specific antigen (PSA) could serve as a better clinically biomarker than total PSA [10–12].

Among the four sialyltransferase families, N-acetylgalactosaminide α-2,6-sialyltransferase (ST6GALNAC) subfamily is in charge of specifically catalyzing the transfer of sialic acid moiety in α-2,6-junction onto GalNAc [13, 14]. In mammals, there are six ST6GALNAC proteins, namely ST6GALNAC1 ~ 6. Of note, onco-foetal sialyl-Tn (sTn) antigen, a truncated O-glycan containing a sialic acid α-2,6 linked to GalNAc α-O-serine/threonine, was elevated in more than 50% high grade PCa patients [15]. This suggests that ST6GALNAC family might be involved in PCa development. However, the key sialyltransferase for the regulation of sialylation in PCa progression and metastasis remains to be identified.

In this study, six ST6GALNAC family members were comprehensively analyzed in PCa samples in two independent PCa datasets (n = 648 in total) and our own cohort containing 64 PCa samples to avoid the potential bias from individual differences of patient samples. ST6GALNAC5 was first identified to be positively associated with tumor progression in PCa. The promoting effects on cell invasion of ST6GALNAC5 were demonstrated in three different PCa cell lines. Moreover, transcriptional factor GATA2 was revealed as an upstream regulator for ST6GALNAC5 in PCa.

## Materials and methods

### Dataset source

The data for gene expression levels and clinical information were downloaded from the TCGA-PRAD (https://portal.gdc.cancer.gov/), NCBI GEO databases (https://www.ncbi.nlm.nih.gov/geo/) for GSE21032 [16], and cBioPortal (http://www.cbioportal.org/), respectively. In sum, 81 normal prostate samples and 648 PCa samples from public datasets were included in the current study. To be noted, some clinicopathological information was missing for certain PCa samples.

### The human protein atlas database

The immunohistochemistry (IHC) results of pathological sections from normal prostate and PCa patients were obtained from The Human Protein Atlas (https://www.proteinatlas.org/).

### Kaplan–Meier survival analysis

The PCa patients were categorized into low and high expression groups for the prognostic survival analysis, according to mRNA levels of six individual ST6GALNAC family members, as well as GATA2-ST6GALNAC5 signature score. The disease-free survival (DFS) was analyzed using Kaplan–Meier survival analysis with log-rank test in PCa patients. p < 0.05 was regarded as statistical significance. The significance of gene relationship with DFS was defined with hazard ratio (HR) and 95% confidence interval (CI) in Cox Proportional-Hazards model.

### Receiver-operating characteristic (ROC) curve analysis

To evaluate the prognostic accuracy of six ST6GALNAC family members and GATA2-ST6GALNAC5 signature, the timeROC package was used to create ROC curves, with which sensitivity as the vertical coordinate and (1-specificity) as the horizontal coordinate, representing the true and false positive rate, respectively. AUC (Area Under ROC Curve) score is an indicator used to evaluate the accuracy of the model, *i.e*., the closer the AUC score is to 1, the higher the accuracy of the model is.

### ***PbCre;Pten***^***F/F***^ PCa mouse model

Mice were bred and maintained under specific pathogen-free conditions in the animal core facilities of Model Animal Research Center, Nanjing University (Nanjing, China). All animal experiments were approved by the Institutional Animal Care and Use Committees (IACUC) at Model Animal Research Center, Nanjing University. By crossbreeding the *PbCre* transgenic mice with the *Pten*-floxed mice [17, 18], we generated *PbCre;Pten*^*F/F*^ mice with a C57BL/6 genetic background, and *Pten*^*F/F*^ was used a control.

### IHC staining of the PCa specimens

64 PCa samples and 10 non-malignant benign prostatic hyperplasia (BPH) samples were from Shanghai Pudong Hospital with the approval by the Ethics Committee of Shanghai Pudong Hospital, Fudan University Pudong Medical Center and the written informed consents of corresponding patients. IHC staining was performed on paraffin-embedded sections with anti-ST6GALNAC5 antibody (1:1000, 16442–1-AP; Proteintech, Rosemont, IL), according to the procedure described previously [19] and visualized by DAB detection kit (DAB-2031; MXB Biotechnologies, Fuzhou, China). Images were acquired by a slide scanner (NanoZoomer 2.0-HT; HAMMATSU, Japan) and analyzed by NDP serve slide distribution and management software (HAMMATSU). The IHC score was the multiplication of the intensity value (0–3) and the positive ratio value (0–3) of the ST6GALNAC5-positive area.

### Gene set enrichment analysis (GSEA)

To evaluate the relationships between meaningful biological processes and ST6GALNAC5 expression, GSEA software (version 4.1.0) from the Broad Institute was used on the C2 KEGG gene sets (v7.2). The nominal p value < 0.05 and false discovery rate (FDR) q < 0.25 were used as cutoff values.

### Gene ontology (GO) enrichment and Kyoto Encyclopedia of Genes and Genomes (KEGG) pathway analyses

The co-expressed genes with ST6GALNAC5 in TCGA-PRAD and GSE21032 datasets were selected by Spearman’s correlation analysis using SangerBox software with │r│ ≥ 0.2 and p < 0.05 as cutoff values. GO enrichment and KEGG pathway analyses were performed using g:Profiler online tool (https://biit.cs.ut.ee/gprofiler/gost/). The enriched GO terms (biological process (BP), cellular components (CC) and molecular function (MF)) and KEGG pathways were presented by a bubble diagram, according to the adjusted p value.

### Protein–protein interaction (PPI) network analysis

The PPI networks were constructed by STRING (https://string-db.org/) and GeneMANIA (http://www.genemania.org). We used the core factors as query proteins to assess PPIs in functional protein association network.

### Plasmid construction

Human ST6GALNAC5 and GATA2 cDNA were cloned into pLVX-3 × Flag-IRES-puromycin and pCDH-4 × HA-T2A-blasticidin, respectively. 2 × Phanta Max Master Mix (P515-01; Vazyme, Nanjing, China) and ClonExpress II One Step Cloning Kit (C112-01; Vazyme) were used for plasmid construction. The primer sequences were shown in Additional file 1: Table S1.

### Cell culture, lentivirus-based transduction and siRNA transfection

Human PCa cell lines (LNCaP, C4-2, 22Rv1, DU145, and PC3) were purchased from the American Type Culture Collection; Human BPH-1 cell line was kindly provided by Dr. Simon Hayward (Vanderbilt University Medical Center, Nashville, TN); 293 T and 293FT cell lines were purchased from Invitrogen (Carlsbad, CA). LNCaP, C4-2, 22Rv1, PC3, and BPH-1 cells were cultured in RPMI 1640 medium, while DU145, 293 T and 293FT cells were maintained in DMEM at 37 °C in a humidified incubator containing 5% CO_2_. Culture media were supplemented with 10% fetal bovine serum (FBS; Gibco, Grand Island, NY), penicillin and streptomycin.pLVX-3 × Flag-ST6GALNAC5-IRES-puromycin or empty vector was co-transfected with pVSV-G and Gag-Pol plasmids into 293FT cells using Lipofectamine 3000 reagent (Invitrogen) to generate lentiviral particles, followed by the transduction to corresponding PCa cells in presence of 8 μg/ml polybrene (H9268; Sigma, St. Louis, MO). siRNAs targeting ST6GALNAC5, GATA2 or negative control siRNA (siNC) were transfected into cells with Lipofectamine RNAiMAX reagent (Invitrogen), according to the manufacturer's instructions. The sequences of siRNAs were listed in Additional file 2: Table S2.

### Western blotting

Cells were lyzed by RIPA buffer with protease inhibitor cocktail tablet (04693116001; Roche, Basel, Switzerland). 10–40 μg protein lysates were loaded to SDS-PAGE and transferred to PVDF membranes (10600021; GE Healthcare, Boston, MA). After the membranes were blocked by 5% non-fatty milk for 30 min, overnight incubation of primary antibodies, including ST6GALNAC5 (1:1000, A17782; ABclonal, Wuhan, China), GATA2 (1:1000, 11103–1-AP; Proteintech), p44/42 MAPK (ERK1/2; 1:1000, #4695; Cell Signaling Technology, Danvers, MA), phospho-p44/42 MAPK (ERK1/2; Thr202/Tyr204; 1:1000, #4370; Cell Signaling Technology), GAPDH (1:20,000, 60004–1-Ig; Proteintech) and mouse monoclonal anti-Flag (1:1000, #F1804; Sigma), was performed at 4 ℃. After incubation with corresponding secondary antibodies, the blots were visualized by SuperSignal West Pico PLUS Chemiluminescent Substrate (34578; Thermo Scientific, Waltham, MA).

### Sulforhodamine B (SRB) assay

3000 PCa cells were seeded in 96-well plates. The starting point was set when PCa cells attached to the bottom. After 24, 48 and 72 h, cells were fixed with 100 μl 10% trichloroacetic acid per well. Afterwards, cells were stained with 4 mg/ml SRB (S1402; Sigma) in 1% acetic acid, followed by the addition of 100 μl 10 mM Tris–HCl to dissolve the SRB. At last, the absorbance was measured at 560 nm by Molecular Devices (SpectraMax M5e; San Jose,　CA).

### Transwell assay

The filter of each 8.0-μm Transwell insert (Jet Biofil, Guangzhou, China) were pre-coated with 100 μl of 250 μg/ml growth factor reduced Matrigel (#356230; Corning). After 2 h, 1 × 10^5^ cells (DU145, C4-2 or 22Rv1) in 100 μl of serum-free medium were seeded in the upper chamber. 600 μl medium with 10% (v/v) FBS was added under the chamber. After 10 h for DU145 cells, 19 h for C4-2 cells, or 24 h for 22Rv1 cells, PCa cells were fixed with 4% formaldehyde and stained with crystal violet staining solution. Images of stained cells underneath the chamber were taken with a microscope (Leica DM6 B, Wetzlar, Germany) and analyzed by ImageJ Software 8 (NIH, Bethesda, MA).

### Genomic mutation analysis

The mutation frequency and mutation type of six ST6GALNAC members in PCa patients were investigated in TCGA-PRAD dataset by cBioPortal online tool (http://www.cbioportal.org/).

### Quantitative real-time PCR (qRT-PCR)

Total RNA was extracted with TRIzol reagent (Invitrogen) and reverse transcribed to cDNA with Hifair II 1st Strand cDNA Synthesis SuperMix (11123ES60; YEASEN, Shanghai, China). qRT-PCR was performed using AceQ qPCR SYBR Green Master Mix (Q341-02; Vazyme) and detected by LightCycler 96 detection system (BioRad, Berkeley, CA). Each experiment was performed in triplicate. Data were analyzed by ΔΔCt method and fold change was determined using *ACTB* for normalization. Primers for qRT-PCR were listed in Additional file 1: Table S1.

### Luciferase assay

Intron 1 of *ST6GALNAC5* gene containing the putative GATA2 binding motif (CGATA) was cloned into pGL3-Basic vector (Promega, Madison, WI) as the ST6GALNAC5-Luc reporter, while the GATA2 binding motif was mutated to CTTTA as described previously [20], using Mut Express II Fast Mutagenesis Kit V2 (C214-02; Vazyme). The primers were listed in Additional file 1: Table S1. 293 T cells in 24-well plates were co-transfected with reporter plasmid (100 ng) and pCDH-4xHA-GATA2 plasmid (0, 25, 50, or 100 ng) using Lipofectamine 2000 reagent (Invitrogen), according to the manufacturer’s instructions. 48 h later, the reporter activity was detected by Firefly Luciferase Reporter Gene Assay Kit (RG005; Beyotime, Shanghai, China). Total protein amount of each sample was used for normalization. Experiments were performed in triplicate.

### Chromatin immunoprecipitation (ChIP) assay

ChIP assay was performed using Magna ChIP A/G chromatin immunoprecipitation kit (#17–10085; Millipore), according to the manufacturer’s protocol. Briefly, 22Rv1 cells were cross-linked by 1% formaldehyde and lysed by cell lysis buffer and nuclear lysis buffer, sequentially. After sonicated by a sonication system (Diagenode, Denville, NJ), the samples were incubated with GATA2 antibody (11103–1-AP; Proteintech) or normal rabbit IgG antibody (#2729; Cell Signaling Technology) and protein A/G magnetic beads at 4 °C overnight with rotation. After washing, the protein/DNA complexes were eluted and reverse cross-linked to free DNA, followed by DNA purification and quantitative PCR. Related primers were listed in Additional file 1: Table S1.

### Calculation of GATA2-ST6GALNAC5 signature score

COX model was used to integrate the prognostic values of GATA2 and ST6GALNAC5 in PCa patients. The formula for calculating the score for each sample was as follows: signature score = β_GATA2_X_GATA2_+β_ST6GALNAC5_X_ST6GALNAC5_. In this formula, X and β indicated the mRNA expression level and the risk coefficient of each gene, respectively.

### Drug sensitivity analysis

Genomics of Drug Sensitivity in Cancer (GDSC) datasets (Release 8.4; http://www.cancerrxgene.org/) and the R package “oncoPredict” were used to predict the targeted therapeutic response of each PCa patient from TCGA-PRAD database. The half-maximal inhibitory concentration (IC_50_) of different drug was estimated by ridge regression. The relevance between drug IC_50_ and ST6GALNAC5 mRNA level was assessed by Spearman’s correlation analysis.

### Statistical analysis

Statistical analysis was performed using GraphPad Prism 8.0 Software (GraphPad Software, La Jolla, CA). Statistical significance of two groups was assessed by Student’s *t* test. Kaplan–Meier survival analysis with log-rank test was used to assess the prognosis differences. p < 0.05 was considered statistically significant.

## Results

### Expression levels of six ST6GALNAC family members in human PCa and normal prostate samples

**Fig. 1:**
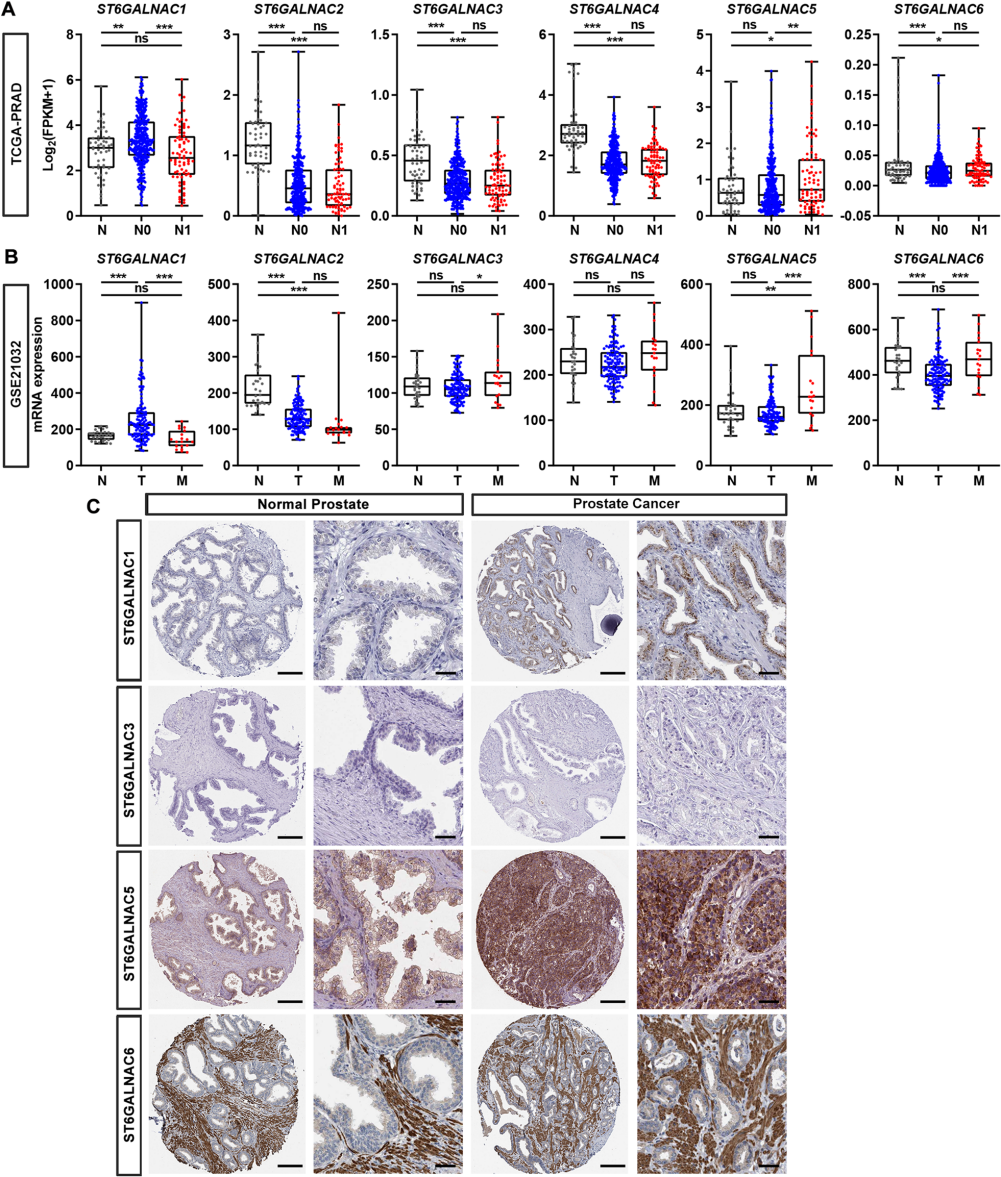
The expression levels of six ST6GALNAC family members in normal prostate and PCa samples. **A** The mRNA expression levels of six ST6GALNAC family members in normal prostate samples (N, n = 52), PCa samples without lymph node metastasis (N0, n = 345) and with lymph node metastasis (N1, n = 80) in TCGA-PRAD dataset. **B** The mRNA levels of six ST6GALNAC family members in normal prostate (N, n = 29), primary PCa (T, n = 131) and metastatic PCa (M, n = 19) in GSE21032 dataset. **C** The representative IHC images of four ST6GALNAC family members in normal prostate and PCa specimens, which were obtained from Human Protein Atlas database. Scale bars, 200 μm for left panels and 50 μm for right panels. *, p < 0.05; **, p < 0.01; ***, p < 0.001; ns, p ≥ 0.05

To explore the role of ST6GALNAC family members in PCa metastasis, we first compared the mRNA levels of six ST6GALNAC family members among normal prostate, primary and metastatic PCa samples in TCGA-PRAD and GSE21032 datasets, respectively. In TCGA-PRAD dataset, ST6GALNAC5 was the only gene consistently up-regulated in PCa samples with lymph node metastatic (N1, n = 80) compared with normal prostate glands (N, n = 52; p < 0.05) and PCa samples without lymph node metastasis (N0, n = 345; p < 0.01; Fig. 1A). To further corroborate the results, we carried out the similar analysis in another dataset GSE21032. As shown in Fig. 1B, ST6GALNAC1 was significantly decreased in metastatic PCa samples, the expression of ST6GALNAC5 mRNA was still higher in metastatic PCa samples (M, n = 19) than that in normal prostate (N, n = 29; p < 0.01) and in primary PCa tissues (T, n = 131; p < 0.001; Fig. 1B). For other five members of ST6GALNAC family, such consistent up-regulation in metastatic PCa was not observed in the above two datasets. Since the Human Protein Atlas database provides the protein distribution on various human tissue sections including tumor tissues, we looked up the available IHC data of ST6GALNAC family members (ST6GALNAC1, 3, 5 and 6) in human normal prostate and PCa specimens (Fig. 1C). The representative IHC data showed that ST6GALNAC1 and ST6GALNAC5 were markedly overexpressed in PCa compared to normal prostates. It was notable that ST6GALNAC5 was mainly expressed in the cytosol of epithelial cells from normal prostate specimens and highly overexpressed in tumor cells from PCa specimens. The mRNA and protein expression data of ST6GALNAC family from public databases containing hundreds of PCa samples indicated a potential correlation between ST6GALNAC5 and PCa metastasis.

### ST6GALNAC5 mRNA level was positively associated with PCa progression

**Fig. 2:**
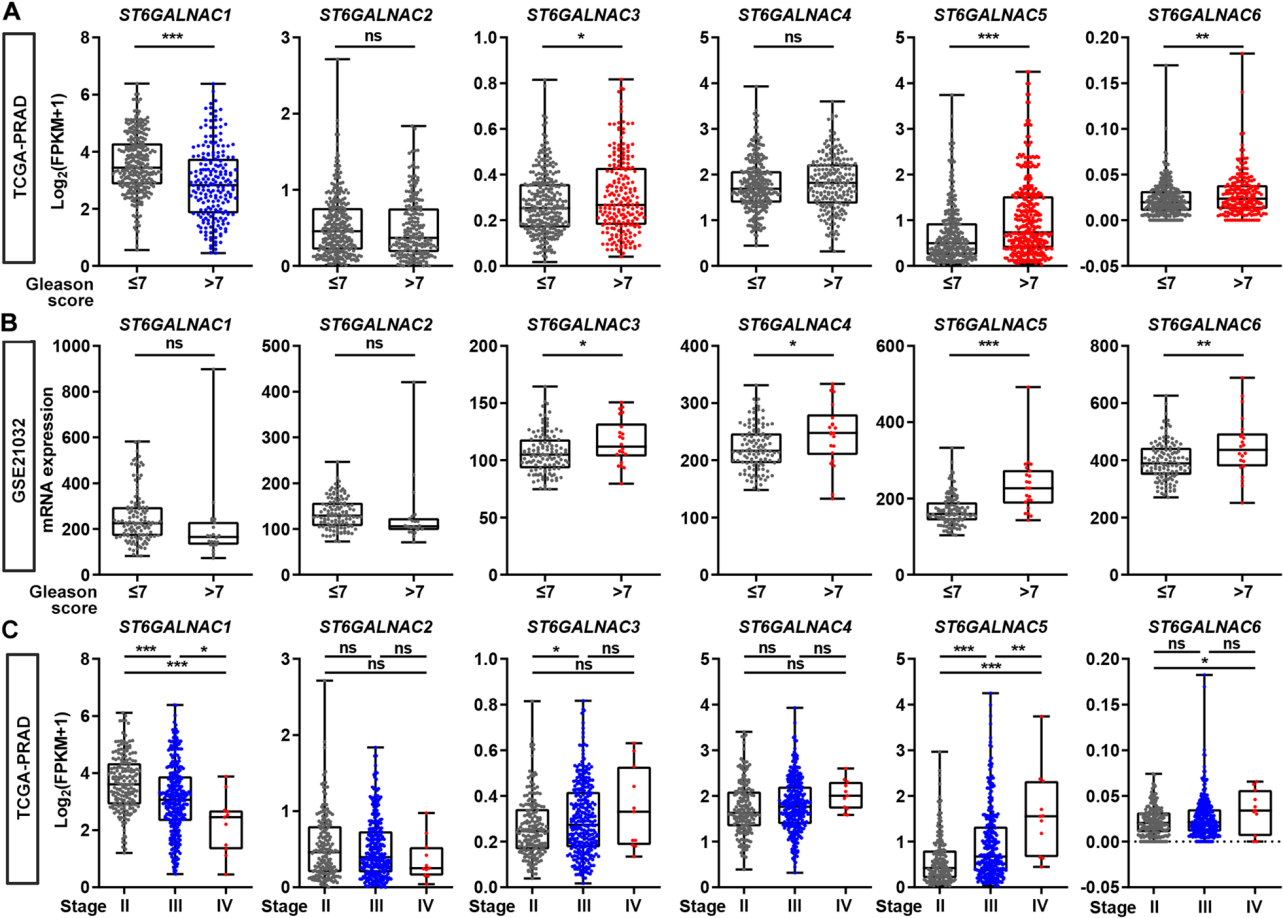
The correlations between each ST6GALNAC family member and clinicopathological parameters in PCa patients. **A**, **B** The mRNA levels of six ST6GALNAC family members were correlated with the pathological Gleason score of PCa patients in TCGA-PRAD dataset (**A**, Gleason score ≤ 7, n = 292; Gleason score > 7, n = 206) and GSE21032 dataset (**B**, Gleason score ≤ 7, n = 117; Gleason score > 7, n = 22). **C** The correlations between mRNA level of each ST6GALNAC family member and tumor stage of PCa patients in TCGA-PRAD dataset. Stage II, n = 187; Stage III, n = 293; Stage IV, n = 11. *, p < 0.05; **, p < 0.01; ***, p < 0.001; ns, p ≥ 0.05

The associations between each ST6GALNAC family member and clinicopathological parameters were then investigated. Gleason score > 7 and ≤ 7 were defined as high- and low-malignancy, respectively. In TCGA-PRAD and GSE21032 datasets, the mRNA expression levels of ST6GALNAC3 (p < 0.05), ST6GALNAC5 (p < 0.001) and ST6GALNAC6 (p < 0.01) were consistently upregulated in PCa patients with high-Gleason score (Fig. 2A, B). In addition, ST6GALNAC5 mRNA level was positively correlated with tumor stages from II to IV (p < 0.01), while ST6GALNAC1 mRNA level was negatively with tumor stages (p < 0.05) in TCGA-PRAD dataset (Fig. 2C). The positive association between ST6GALNAC5 and PCa progression was thus suggested.

### ST6GALNAC5 was a potential poor prognostic indicator in PCa patients

**Fig. 3:**
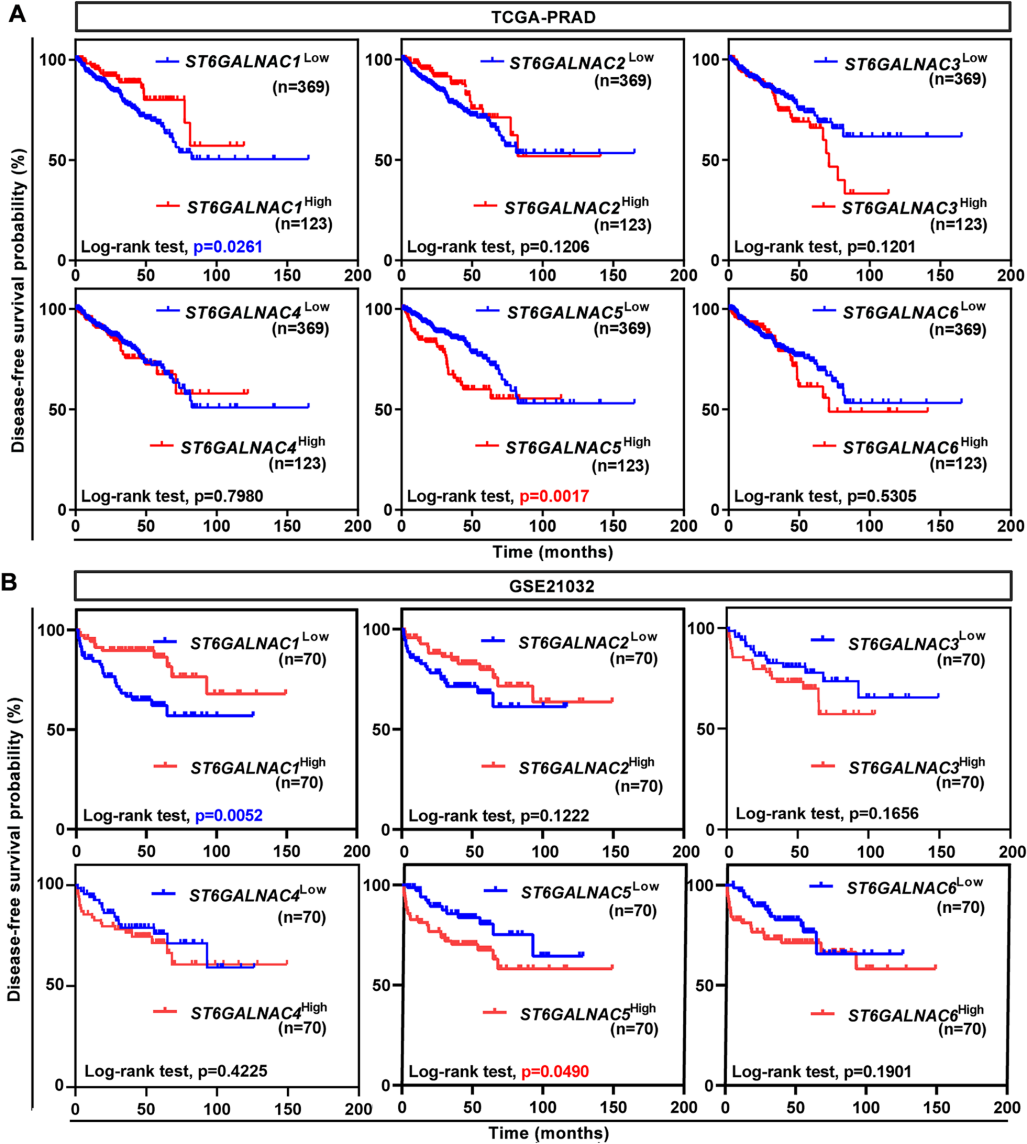
The prognostic values of six ST6GALNAC family members in PCa patients. **A**, **B** Kaplan–Meier plot of DFS in PCa patients from TCGA-PRAD dataset (**A**) and GSE21032 dataset (**B**) stratified by mRNA expression level of each ST6GALNAC member

The effect of ST6GALNAC family members on patient survival was further assessed in PCa. DFS status was plotted stratifying by mRNA levels of each ST6GALNAC members. The Kaplan–Meier survival analysis revealed that high level of ST6GALNAC5 mRNA correlated with poor outcome in TCGA-PRAD dataset (p = 0.0017, HR = 1.943, 95% CI 1.197 to 3.153; Fig. 3A) and in GSE21032 dataset (p = 0.0490, HR = 1.954, 95% CI 1.016 to 3.759; Fig. 3B); meanwhile ST6GALNAC1-high expression group showed longer DFS duration than ST6GALNAC1-low group in TCGA-PRAD dataset (p = 0.0261, HR = 0.5392, 95% CI 0.3394 to 0.8565; Fig. 3A) and in GSE21032 dataset (p = 0.0052, HR = 3795, 95% CI 0.1971 to 0.7307, Fig. 3B). The ROC curve analysis revealed that ST6GALNAC5 showed the best discriminative ability for DFS of 3-year (AUC score = 0.880) and 5-year (AUC score = 0.846) among the ST6GALNAC members in GSE21032 dataset (Additional file 3: Fig. S1). These results indicated that ST6GALNAC5 was a promising prognostic biomarker in PCa patients.

### The overexpression of ST6GALNAC5 in a PCa mouse model and PCa patients from our cohort

**Fig. 4:**
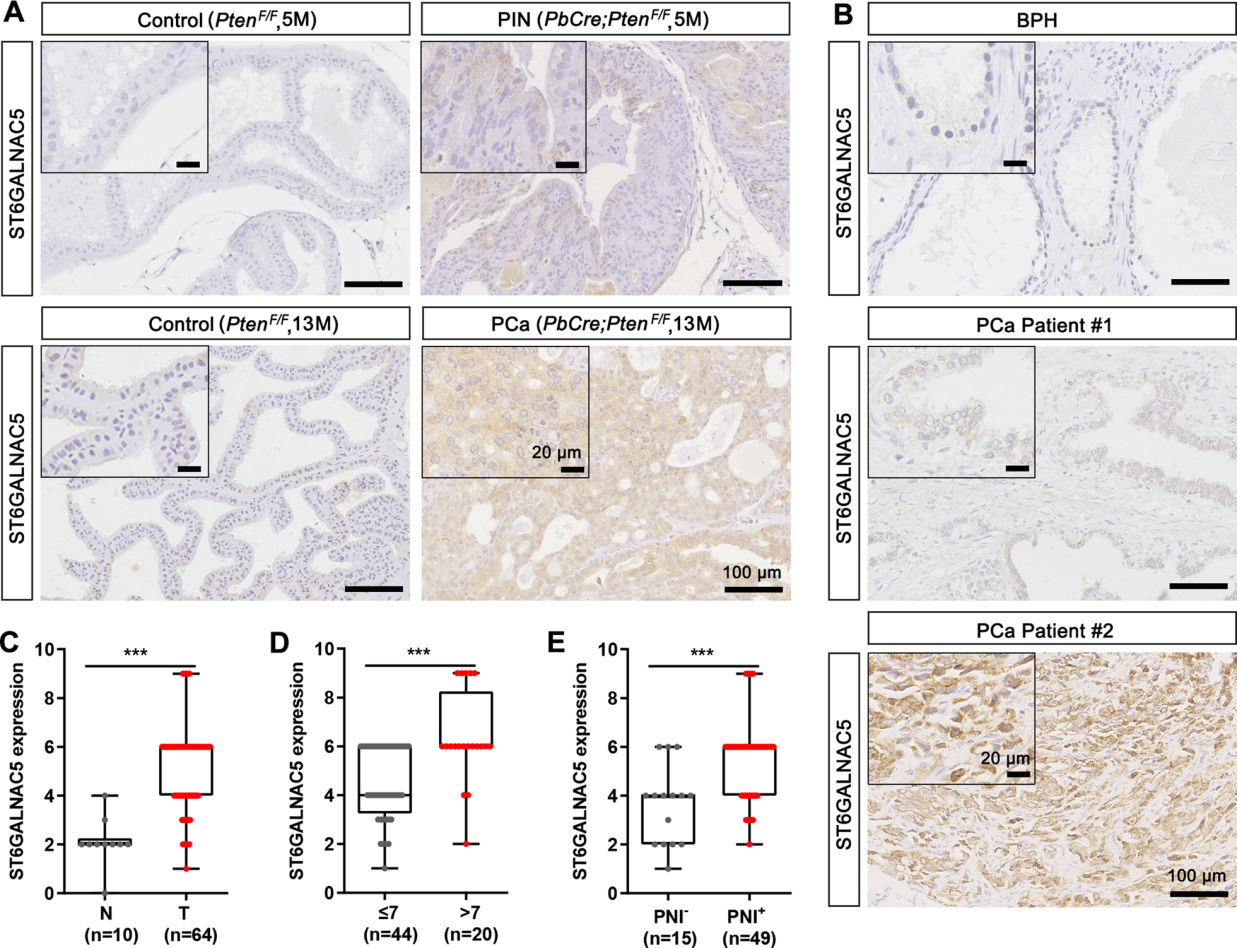
Overexpressed ST6GALNAC5 in mouse and human PCa specimens from our cohort. **A** The representative IHC images of ST6GALNAC5 in the normal prostate from a 5-month-old *Pten*^*F/F*^ mouse (control), PIN from a 5-month-old *PbCre;Pten*^*F/F*^ mouse, normal prostate from a 13-month-old *Pten*^*F/F*^ mouse (control), and PCa from a 13-month-old *PbCre;Pten*^*F/F*^ mouse. **B** The representative IHC images of ST6GALNAC5 in one non-malignant human BPH and two PCa specimens. Scale bar, 100 μm; inset, 20 μm. **C** The quantification on ST6GALNAC5 protein levels in BPH samples (N, n = 10) and PCa samples (T, n = 64) by IHC staining. **D** The quantification on ST6GALNAC5 protein levels in PCa samples with Gleason score ≤ 7 (n = 44) and PCa with Gleason score > 7 (n = 20) by IHC staining. **E** The quantification on ST6GALNAC5 protein levels in PCa samples with PNI (n = 49) and without PNI (n = 15) by IHC staining. ***, p < 0.001

To validate whether ST6GALNAC5 level is indeed elevated in PCa development, we first examined the ST6GALNAC5 expression in a well-known genetic modified PCa mouse model, *PbCre;Pten*^*F/F*^. In the control *Pten*^*F/F*^ mouse (5-month old), ST6GALNAC5 was barely detected in normal prostate epithelial cells; while in the *PbCre;Pten*^*F/F*^ mouse with prostatic intraepithelial neoplasia (PIN; 5-month old), ST6GALNAC5 was detected in the cytosol of some prostate epithelial cells. Moreover, in the control *Pten*^*F/F*^ mouse (13-month old), ST6GALNAC5 was barely detectable; but in the *PbCre;Pten*^*F/F*^ mouse with PCa (13-month old), ST6GALNAC5 intensity was markedly increased in almost all PCa cells (Fig. 4A). The clinical relevance of ST6GALNAC5 was further confirmed in PCa samples from our cohort, containing 64 PCa specimens and 10 BPH specimens as the non-malignant controls. As shown in Fig. 4B, C, ST6GALNAC5 was weakly detected in BPH samples, but overexpressed in PCa specimens (p < 0.001). We also observed that ST6GALNAC5 protein levels in PCa samples with Gleason score > 7 (n = 20) were much higher than those in PCa samples with Gleason score ≤ 7 (n = 44, p < 0.001; Fig. 4D). Perineural invasion (PNI) is a complex process of neoplastic invasion to nerves, which is recognized as a significant route for metastatic spread of PCa cells [21]. PNI is frequently detected in up to 75% of surgically resected PCa specimens, with adverse pathological features, increased risk of bone metastases, elevated biochemical recurrence rates, and reduced overall survival [22–25]. Notably, we found that ST6GALNAC5 protein levels were significantly elevated in the PNI-positive PCa samples, compared to the PNI-negative one (p < 0.001; Fig. 4E). Taken together, ST6GALNAC5 overexpression was confirmed in both mouse PCa model and human PCa patients and was significantly associated with PCa progression.

### Function enrichment and pathway analysis of ST6GALNAC5 in PCa patients

**Fig. 5:**
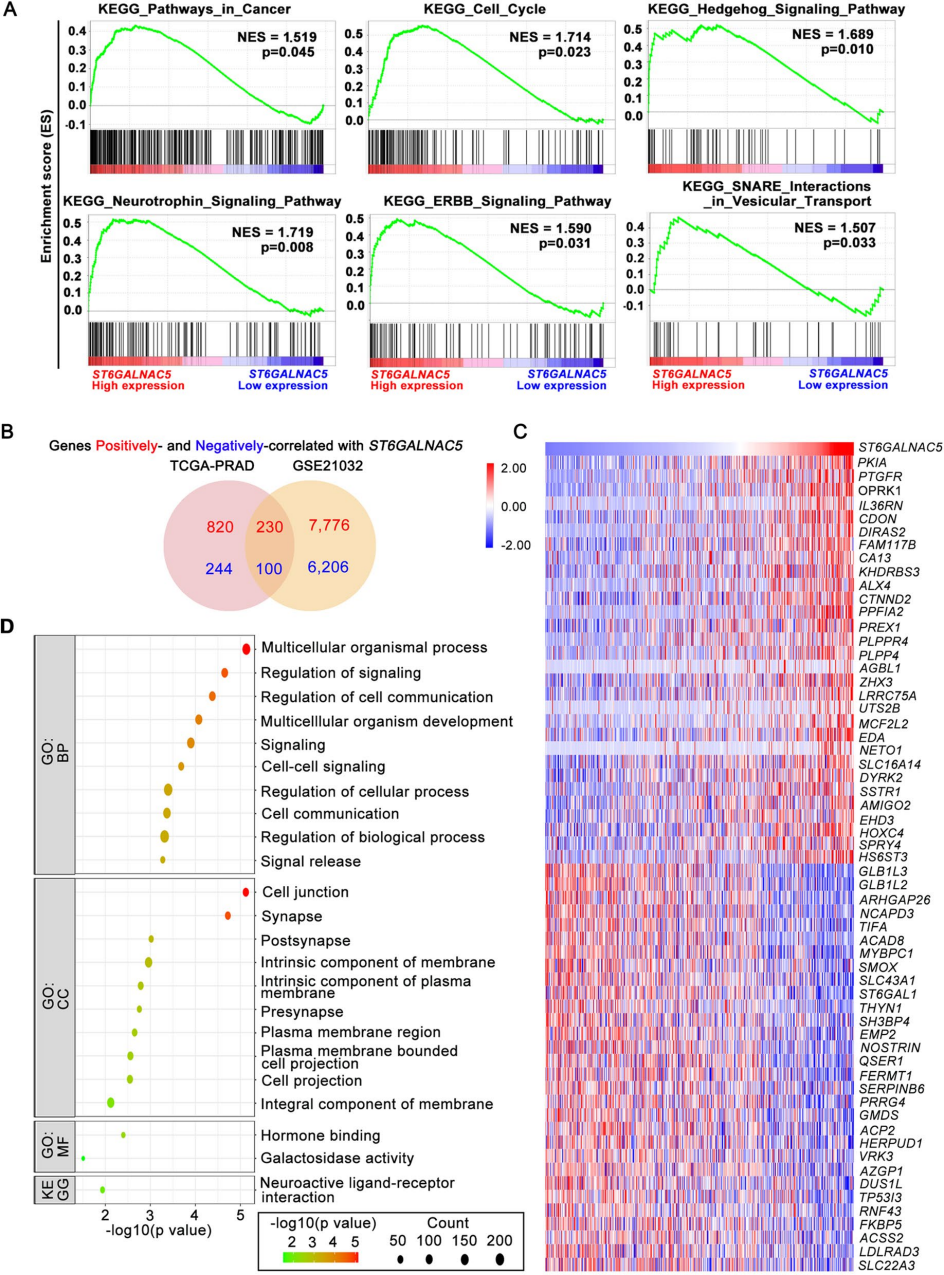
Potential involvement of ST6GALNAC5 in PCa metastasis. **A** GSEA on ST6GALNAC5 in TCGA-PRAD dataset. **B** The genes positively- and negatively-correlated with ST6GALNAC5 in TCGA-PRAD and GSE21032 datasets by Venn diagram. **C** The heatmap of top 30 genes positively- and negatively-correlated with ST6GALNAC5 in TCGA-PRAD dataset. **D** GO enrichment and KEGG analyses of ST6GALNAC5-related pathways and functions. *BP* biological processes, *CC* cellular components, *MF* molecular function

To investigate the functions and related pathways of ST6GALNAC5 in PCa patients, we performed GSEA in TCGA-PRAD dataset. The results showed that ST6GALNAC5 mRNA level was associated with the genesets related to cancer, cell cycle, Hedgehog signaling, neurotrophin signaling, ERBB signaling, and SNARE interaction (Fig. 5A).

Next, we screened out ST6GALNAC5-associated genes (with similar or reverse expression patterns) in both TCGA-PRAD and GSE21032 datasets. 230 genes were positively correlated with ST6GALNAC5, while 100 genes were negatively correlated with ST6GALNAC5 (Fig. 5B). The top 30 positively/negatively associated genes with ST6GALNAC5 in TCGA-PRAD dataset were shown in Fig. 5C. GO enrichment and KEGG analyses were performed on these consistently changed genes. GO enrichment showed that cell junction was identified in the CC category, and regulation of cell communication, cell–cell signaling, and cell communication were identified in the BP category. KEGG pathway analysis showed that neuroactive ligand-receptor interaction pathway was related to ST6GALNAC5 (Fig. 5D).

PPI network of ST6GALNAC5 was then constructed using STRING and GeneMANIA programs (Additional file 4: Fig. S2). As expected, several glyco-metabolism-related proteins, such as ST3GAL1, ST3GAL2, and ST8SIA5, were identified by both programs. We also noticed HBEGF protein, which was reported to be involved in breast cancer cell metastasis [26].

Overall, the above results suggested that ST6GALNAC5 may regulate the cell junction, differentiation and metastasis in PCa.

### ST6GALNAC5 promoted PCa cell invasion

**Fig. 6:**
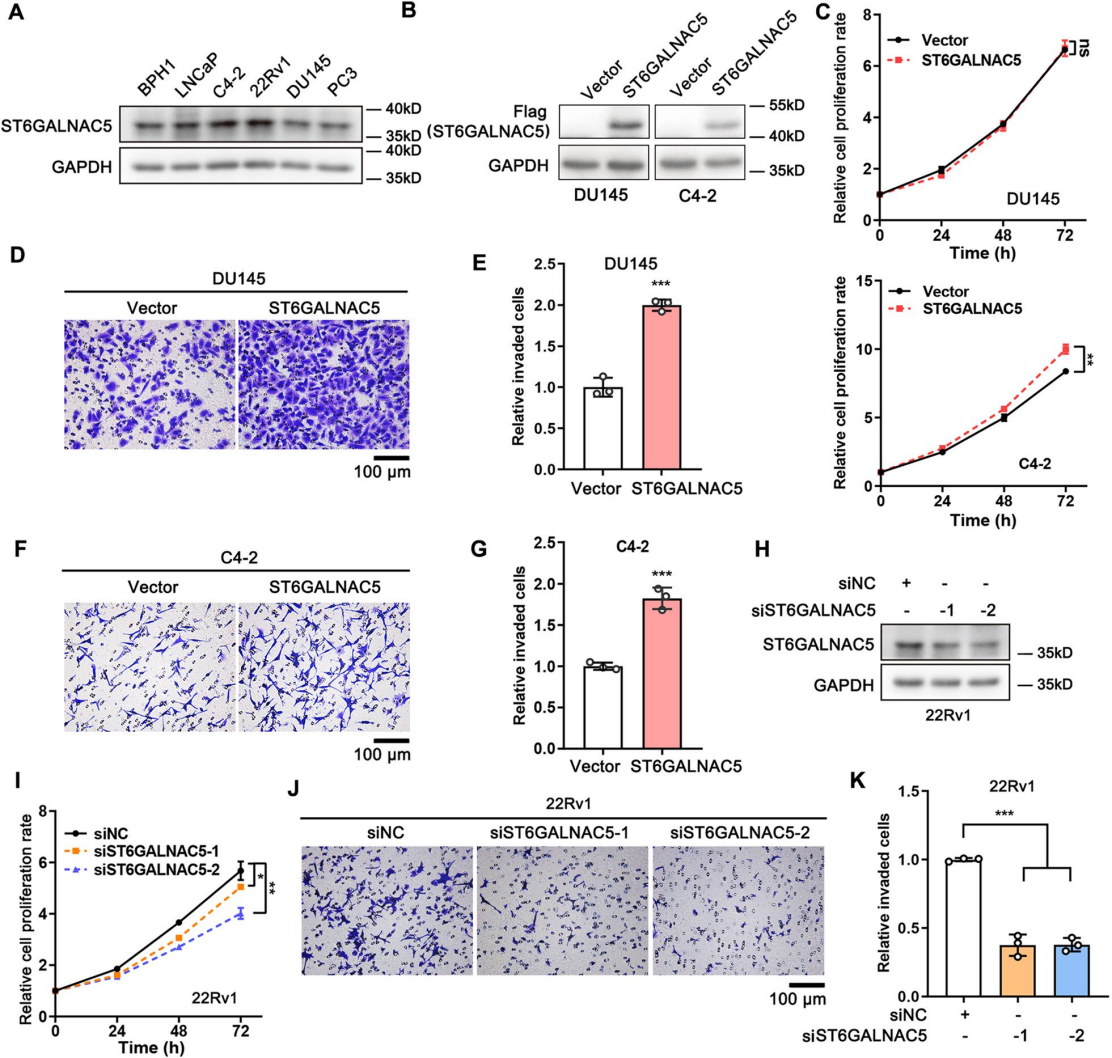
ST6GALNAC5 promoted the invasion of PCa cells. **A** ST6GALNAC5 protein levels in non-malignant BPH-1 and multiple PCa cell lines by Western blotting. **B** The stable ectopic expression of ST6GALNAC5 in DU145 and C4-2 PCa cells. **C** Effects of ST6GALNAC5 overexpression on the proliferation of DU145 and C4-2 cells. **D**, **E** The representative images (**D**) and quantification (**E**) of cell invasion for ST6GALNAC5 overexpressed and control DU145 cells by transwell assay. Scale bar, 100 µm. **F**, **G** The representative images (**F**) and quantification (**G**) of cell invasion for ST6GALNAC5 overexpressed and control C4-2 cells by transwell assay. Scale bar, 100 µm. **H** Knockdown ST6GALNAC5 by two siRNAs in 22Rv1 cells. **I** Effect of ST6GALNAC5 knockdown on the proliferation of 22Rv1 cells. **J**, **K** The representative images (**J**) and quantification (**K**) of cell invasion for ST6GALNAC5-knockdown and siRNA control (siNC) 22Rv1 cells by transwell assay. Scale bar, 100 µm. *, p < 0.05; **, p < 0.01; ***, p < 0.001; ns, p ≥ 0.05

To determine the effects of ST6GALNAC5 on PCa aggressiveness, PCa cell proliferation and invasion were assessed by SRB and Transwell assays, respectively. We examined the endogenous expression levels of ST6GALNAC5 in one BPH and five PCa cell lines (Fig. 6A). ST6GALNAC5 was expressed relatively lower in non-malignant BPH-1 cells as well as in DU145, PC3 and C4-2 PCa cells, and the highest expression of ST6GALNAC5 was observed in 22Rv1 cells. Thus, DU145 and C4-2 cells were chosen for stable overexpression of Flag-tagged ST6GALNAC5 (Fig. 6B). Although the ectopic expression of ST6GALNAC5 significantly increased C4-2 cell proliferation (p < 0.01), such proliferation-promoting effect was not detected in DU145 cells (Fig. 6C). Notably, ST6GALNAC5 overexpression significantly and consistently increased the cell invasion by ~ 2.0-fold comparing to the vector control cells in DU145 cells (p < 0.001; Fig. 6D, E) and C4-2 cells (p < 0.001; Fig. 6F, G).

On the other hand, 22Rv1 cells were selected to knockdown ST6GALNAC5 expression by RNA interfering. Two siRNAs targeting two different regions of ST6GALNAC5 were used to exclude off-target effects (Fig. 6H). ST6GALNAC5 depletion not only reduced cell proliferation (p < 0.05; Fig. 6I), but also decreased the cell invasiveness (p < 0.001; Fig. 6J, K). These results implicated that ST6GALNAC5 was indispensable for the invasion of PCa cells.

### ST6GALNAC5 was positively regulated by transcription factor GATA2 in PCa cells

In order to address why ST6GALNAC5 was overexpressed in PCa, we investigated the genetic alterations of *ST6GALNAC* family members in PCa patients in three PCa datasets, including MSKCC/DFC, TCGA-PRAD (Firehose Legacy) and MCTP, by cBioPortal online tool. Even in MSKCC/DFC dataset, the overall genetic alteration frequency of whole *ST6GALNAC* family was only 9.84% (Additional file 5: Fig. S3A). There were only 2.4% of PCa patients with the genetic alterations of *ST6GALNAC5*, the majority of which was deep deletion (Additional file 5: Fig. S3B). The relatively low frequency of genetic changes in *ST6GALNAC5* implied that other factors might contribute to its overexpression in PCa.

**Fig. 7:**
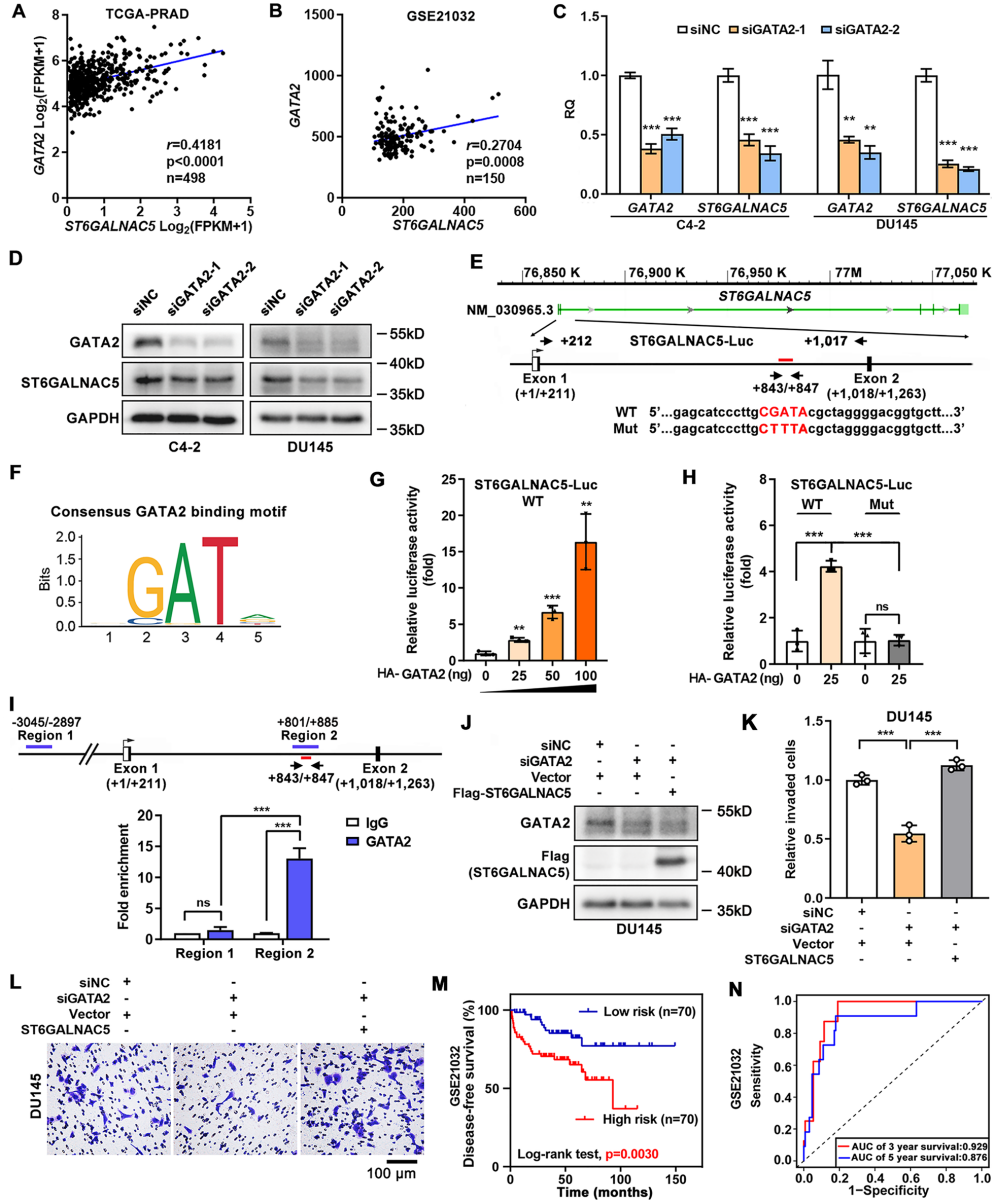
GATA2 mediated PCa cell invasion via ST6GALNAC5. **A**, **B** The correlation between ST6GALNAC5 and GATA2 expression in TCGA-PRAD dataset (**A**) and GSE21032 dataset (**B**). **C** The mRNA levels of GATA2 and ST6GALNAC5 in C4-2 and DU145 cells transfected with siRNAs targeting GATA2 by qRT-PCR. **D** The protein levels of GATA2 and ST6GALNAC5 in C4-2 and DU145 cells transfected with siRNAs targeting GATA2 by Western blotting. **E** Genomic structure of *ST6GALNAC5*. Boxes, the locations of exons of *ST6GALNAC5*. Schematics of a putative GATA2 binding site (red line) within intron 1. The ST6GALNAC5 genomic region (+ 212 to + 1017; WT) containing a GATA2 binding motif (in red) was inserted to pGL3-Basic vector as ST6GALNAC5-Luc. The ST6GALNAC5-Luc mutation (Mut) was generated using the “CTTTA” sequence to replace the GATA2 motif. **F** The putative binding site of transcriptional factor GATA2 on the *ST6GALNAC5* promotor. **G** Relative luciferase activity of wild-type (WT) ST6GALNAC5-Luc reporter in 293 T cells transfected with HA tagged-GATA2 at different concentrations. **H** Relative luciferase activity of ST6GALNAC5-Luc reporters (WT and Mut) in 293 T cells transfected with HA tagged-GATA2. **I** ChIP assay for GATA2 recruitment to *ST6GALNAC5* gene. A schematic diagram of *ST6GALNAC5* gene for ChIP assay was shown in upper panel. GATA2 enrichments on the *ST6GALNAC5* locus in 22Rv1 cells, with IgG antibody as a negative control (lower panel). **J** The effects of siRNAs targeting GATA2 on ST6GALNAC5 protein levels in parental and Flag-tagged ST6GALNAC5-overexpressed DU145 cells. **K**, **L** The quantification (**K**) and representative images (**L**) of cell invasion for DU145 cells treated with GATA2 siRNA alone or combined with ST6GALNAC5 overexpression by transwell assay. Scale bar, 100 µm. **M** Kaplan–Meier plot of DFS in PCa patients from GSE21032 dataset stratified by the risk score of GATA2-ST6GALNAC5 signature. **N** ROC curve analysis on the risk score of GATA2-ST6GALNAC5 signature in PCa patients from GSE21032 dataset. **p < 0.01, ***, p < 0.001, ns, p ≥ 0.05

Hence, the transcription (co)factors which were co-expressed with ST6GALNAC5 in both TCGA-PRAD and GSE21032 datasets were further analyzed (Fig. 5B). We identified mRNA level of GATA2 gene, the important transcription factor involved in PCa metastasis and castration resistance [27, 28], was significantly and positively associated with ST6GALNAC5 mRNA level in TCGA-PRAD (p < 0.0001, r = 0.4181; Fig. 7A) and GSE21032 (p = 0.0008, r = 0.2704; Fig. 7B).

To test whether GATA2 was an upstream regulator of ST6GALNAC5 gene, we knocked down GATA2 expression by two independent siRNAs in C4-2 and DU145 cells. GATA2 depletion significantly downregulated ST6GALNAC5 expression at both mRNA (Fig. 7C) and protein levels (Fig. 7D) in PCa cells. Next, to dissect how GATA2 regulated ST6GALNAC5 gene expression, we performed bioinformatics analysis on the JAPSPAR website (http://jaspar.genereg.net/) and successfully identified a putative GATA2 binding site within intron 1 of *ST6GALNAC5* gene (Fig. 7E, F). The ST6GALNAC5-Luc reporter containing the predicted GATA2 binding site was generated, and ST6GAL5 genomic region inserted to the vector was shown in Fig. 7E. By luciferase assay, we found that GATA2 increased the transcriptional activity of ST6GALNAC5 promoter in a dose-dependent manner (Fig. 7G); however, the mutated reporter lost its response to GATA2 overexpression significantly (p < 0.001) (Fig. 7H). Furthermore, GATA2 binding to intron 1 of *ST6GALNAC5* gene was demonstrated by ChIP assay. GATA2 protein was recruited to Region 2 (+ 801 to + 885), containing a predicted GATA2 binding motif (+ 843 to + 847), but not to Region 1 (-3045 to -2897), without such motif (Fig. 7I). To verify ST6GALNAC5 as the downstream target of GATA2 in PCa cells, we ectopically expressed ST6GALNAC5 in GATA2-depleted DU145 cells (Fig. 7J). As shown in Fig. 7K, L, GATA2 knockdown in DU145 cells significantly reduced the invasion capacity of cancer cells (p < 0.001). Introduction of ST6GALNAC5 significantly reversed the compromised cancer cell invasiveness in DU145 cells with GATA2 deficiency, indicating that ST6GALNAC6 was an important target to GATA2 in cancer cell invasion.

The clinical relevance of GATA2 gene in PCa was further investigated. In GSE21032 dataset, GATA2 mRNA level was significantly elevated in PCa samples with metastasis (p < 0.001) compared to those without metastasis (Additional file 6: Fig. S4A); GATA2 mRNA level was also positively associated with Gleason score (Additional file 6 Fig. S4B). Additionally, GATA2 predicted shorter DFS duration in PCa patients with p = 0.0106 (Additional file 6: Fig. S4C). The AUC scores of GATA2 for DFS of 3-year and 5-year were 0.872 and 0.826, respectively (Additional file 6: Fig. S4D). Of note, when we used the GATA2-ST6GALNAC5 signature, better prediction for patient outcome of DFS (p = 0.0030; Fig. 7M) and discriminative ability for 3-year (AUC score = 0.929) and 5-year DFS (AUC score = 0.876; Fig. 7N) were observed, comparing to GATA2 and ST6GALNAC5 individual gene. Overall, ST6GALNAC5 was positively regulated by GATA2 and the GATA2-ST6GALNAC5 signature might be a promising prognostic biomarker in PCa patients.

### Relationship between ST6GALNAC5 expression and drug sensitivity

**Fig. 8:**
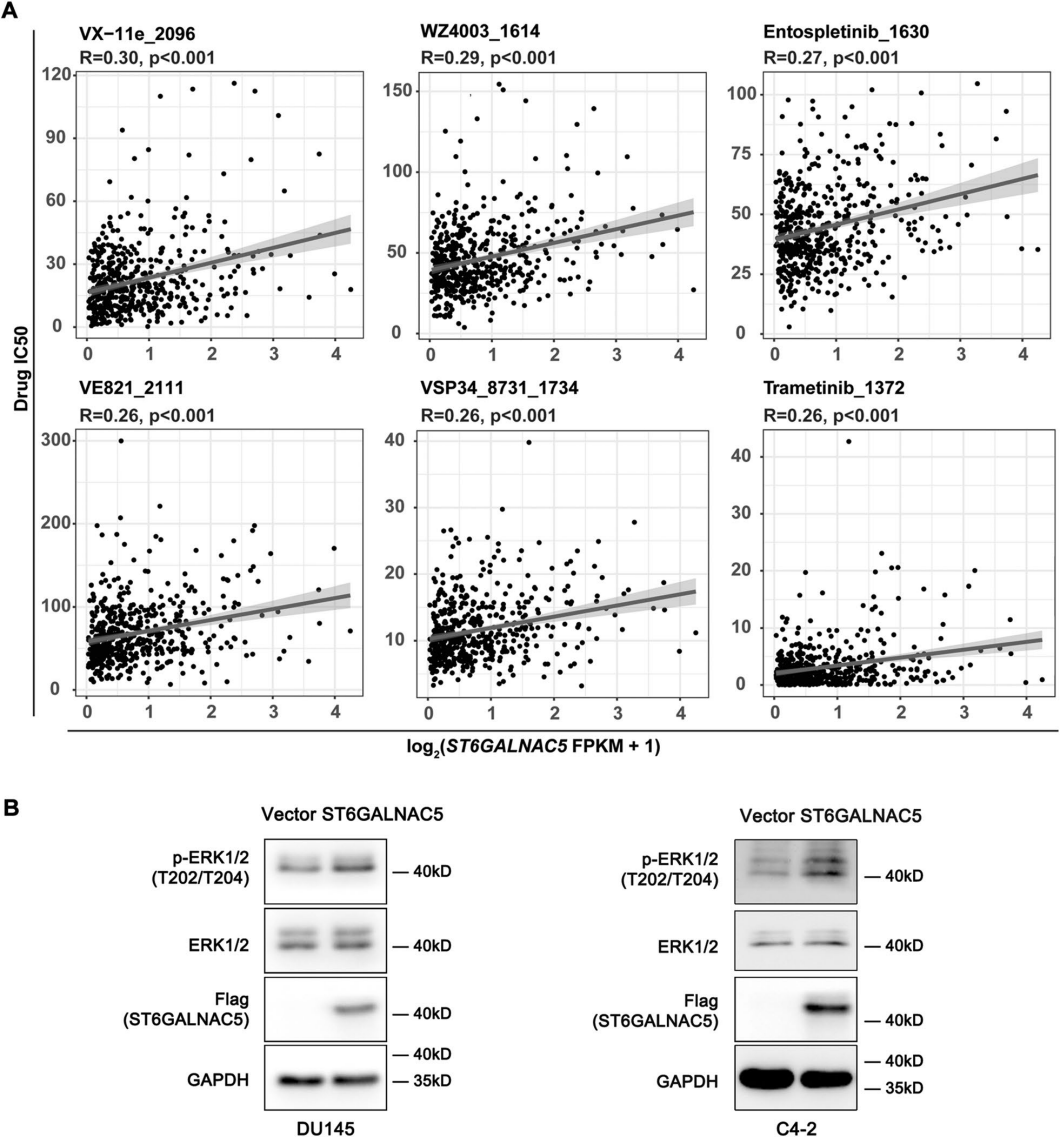
**A** Drug sensitivity analysis on relationship between the predicted drug IC_50_ value and ST6GALNAC5 mRNA level. **B** The levels of ERK proteins in DU145 and C4-2 cells with ST6GLNAC5 overexpression by Western blotting

Altered gene expression may cause changes to drug response in cancer patients. To explore whether ST6GALNAC5 expression could influence drug sensitivity in PCa patients, we performed a correlation analysis between ST6GALNAC5 expression level from TCGA-PRAD and predicted IC_50_ value of different drugs by R package “oncoPredict” based on GDSC database, which has characterized > 1000 human cancer cell lines and screened them with 621 compounds targeting 24 pathways to correlate the compound sensitivity data with genomic datasets for molecular features associated with drug sensitivity and resistance [29]. Significant positive associations (p < 0.001) between ST6GALNAC5 mRNA expression and the resistance to six drugs were predicted as follow, VX-11e (R = 0.30), WZ4003 (R = 0.29), Entospletinib (R = 0.27), VE821 (R = 0.26), VSP34_8731 (R = 0.26), and Trametinib (R = 0.26) (Fig. 8A). Since VX-11e (an ERK inhibitor), Entospletinib (a Syk inhibitor), and Trametinib (a MEK inhibitor) have been reported to inhibit the phosphorylation of ERK [30–33], phospho-ERK levels in PCa cells with different expression levels of ST6GALNAC5 were tested. We found ST6GALNAC5 overexpression increased ERK phosphorylation in DU145 and C4-2 cells (Fig. 8B). These results indicated that the predicted sensitivity of targeted therapeutic drugs may help us uncover the downstream signalings of ST6GALNAC5 and in turn benefit the drug selection for PCa patients.

## Discussion

Aberrant changes of sialylation on glycoproteins and metabolites have been frequently reported in various cancer types in past decades, which are associated with the altered expression levels and activities of sialyltransferases. Given that PCa patients with metastatic lesions still experience poor prognosis, more reliable diagnostic/prognostic biomarkers are urgently required. Some sialylated forms of glycans were reported to be associated with PCa progression and metastasis, including sialyl Lewis antigens and sialyl-Tn antigen [11], suggesting potential roles of the related sialyltransferases in PCa metastasis. Herein, we performed a comprehensive analysis on six ST6GALNAC family members, which are responsible for the transfer of sialic acid moiety to α-2,6-junction onto GalNAc, in two independent PCa cohorts containing PCa samples with metastasis (n = 99) and without metastasis (n = 476) in total. We successfully identified ST6GALNAC5 as a gene positively associated with PCa aggressiveness and metastasis, heralding poor clinical outcomes in PCa patients.

ST6GALNAC5, also known as ST6GalNAc V or SIAT7E, is specifically expressed in both human and mouse brain tissues, and functions as a synthase for 0-series ganglioside GD1α from GM1b, without changing α2,6-linked sialic acid-containing glycoproteins [26, 34, 35]. Its overexpression was reported to increase GD1α ganglioside expression on the surface of breast cancer cells, reduce the adhesion of breast cancer cells to blood–brain barrier and facilitate brain-specific metastasis [36, 37]. In addition, the expression of ST6GALNAC5 was also required for epithelial-to-mesenchymal transition (EMT) of MDA-MB-231 breast cancer cells [38]. ST6GALNAC5 could also enhance the anchorage-independent growth of MDCK cells by activating the HGF/MET signaling [39]. Hence, its tumor-promoting role was indicated. However, ST6GALNAC5 was found to be downregulated in glioma. Its forced expression in glioma cells inhibited the cell invasiveness in vitro and tumorigenicity in vivo [35, 40]. Interestingly, ST6GALNAC5 stably overexpressed U373MG glioma cells showed the decrease of GM1b, Gb3 and Gb4 gangliosides, and the increase of GM2α, but not GD1α. The context-dependent role of sialyltransferases is not unforeseen. For example, the reduction of ST3GAL6 was frequently detected in colorectal cancer and lung adenocarcinoma [41, 42]; while ST3GAL6 overexpression promoted bladder cancer cell invasion and homing/survival of multiple myeloma cells [8, 43]. These evidences suggest that the important role of ST6GALNAC5 in tumor metastasis, but promoting or inhibitory effect is highly dependent on cancer cell context. The lipidomic analysis may be performed to further analyze the changes of various gangliosides in response to dysregulation of ST6GALNAC5 in the specific tumor cells.

How ST6GALNAC5 is regulated in cancer development is under way. In the process of EMT, TGFβ1 upregulated ST6GALNAC5 in A549 lung adenocarcinoma cells, while another EMT negative regulator miR-200b downregulated ST6GALNAC5 through directly targeting its 3’UTR [39]. The translation efficiency of ST6GALNAC5 mRNA was also tightly regulated by its m^6^A modification and read by the m^6^A reader YTHDF3 in brain-specific metastatic breast cancer cells [44]. Though the mechanism of its downregulation in cancer is not well-documented, its promoter methylation was suggested in HPV( +) cervical cancer [45, 46]. Herein, we identified that GATA2 transcription factor could positively regulate ST6GALNAC5 expression in PCa cells. As one of the members of GATA family (GATA1 ~ 6), GATA2 plays a central role in development of hematopoietic and genitourinary systems [47–50]. Recently, GATA2 overexpression was frequently reported to be associated with PCa aggressiveness, indicating its potential as a poor prognostic biomarker in PCa patients [26, 27]. GATA2 could promote PCa progression in an AR-dependent manner, embodied in facilitating the expression of AR and AR target gene, which was also considered to function as a pioneer factor [51, 52]. In addition, GATA2 was involved in the development of CRPC and metastatic PCa [53] through directly upregulating a battery of target genes, such as BMP6, EZH2, FOXM1 and IGF2, which were associated with PCa metastasis and taxane resistance [26, 54, 55]. Our current study indicated that ST6GALNAC5 may also contribute to the oncogenic effects of GATA2 to promote PCa cell invasion.

The bioinformatics data from TCGA-PRAD and GSE21032 databases in Fig. 5 did show that several pathways including cell junction were associated with ST6GALNAC5 expression level. Considering that ST6GALNAC5 functions as a synthase for 0-series ganglioside GD1α from GM1b, we speculate that GD1α and/or its related downstream gangliosides, which reside on cell membrane through their lipid chains, may affect receptor tyrosine kinases or other membrane proteins through microdomain formation [56]. In this study, we found that overexpression of ST6GALNAC5 activated MAPK/ERK1/2 pathway in prostate cancer cells. AP-1/c-Fos and ETS-1, as the transcriptional factors activated by MAPK/ERK1/2 pathway, have been reported for the upregulation of OPRK1 and CEACAM1 [57, 58], which were also identified in our “Cell Junction” list. Therefore, we speculate that ST6GALNAC5 overexpression may indirectly induce cell junction gene expression at transcription level, at least through ERK1/2 signaling pathway.

ST6GALNAC family members have been implicated in cancer development and therapy resistance. The ectopic expression of ST6GALNAC1 in PCa cells reduced tumor growth, but did not affect metastasis in vivo [15]; while ST6GALNAC2 was reported to function as a cancer metastasis suppressor in breast cancer [59], which was consistent with its lower expression in PCa than normal prostate samples (Fig. 1). Moreover, previous studies have showed that ST6GALNAC1 was associated with chemotherapeutic resistance in gastric cancer and ST6GALNAC6 might affect drug resistance in ovarian cancer [60, 61]. The potential association between ST6GALNAC5 expression and drug sensitivity in PCa was thus investigated. Our results showed the higher level of ST6GALNAC5 mRNA was positively correlated with the higher predicted resistance to several drugs, including an ERK inhibitor, a Syk inhibitor, and a MEK inhibitor. Consistently, the level of phosphorylated ERK, a downstream target of these three drugs, had a notable positive correlation with ST6GALNAC5 expression in PCa cells, implying an adjunctive ST6GALNAC5-targeted therapy for PCa patients.

## Conclusions

In the present study, we identified that ST6GALNAC5 expression level was positively associated with PCa progression, indicating its potential as a prognostic biomarker for PCa patients. Moreover, transcription factor GATA2 was first revealed to upregulate ST6GALNAC5 gene expression and to promote PCa cell invasion.

## Supplementary Information

**Additional file 1: Table S1.** The primers for plasmid construction, qRT-PCR and ChIP.

**Additional file 2: Table S2.** The sequences of siRNAs.

**Additional file 3: Figure S1. **ROC curve analysis on six ST6GALNAC family members in PCa patients from GSE21032 dataset.

**Additional file 4: Figure S2. **The PPI analyses of ST6GALNAC5 in PCa samples. PPI network of ST6GALNAC5 was constructed by STRING (A) and GeneMANIA (B) programs.

**Additional file 5: Figure S3. **The genetic alterations of ST6GALNAC family members in PCa samples were analyzed in plot (A) and in individual cases (B) from the cBioPortal online database.

**Additional file 6: Figure S4. **The association analysis on GATA2 mRNA expression with clinicopathological parameters of PCa patients in GSE21032 dataset. (A) The mRNA level of GATA2 in normal prostate (N, n=29), primary PCa (T, n=131) and metastatic PCa (M, n=19) in GSE21032 dataset. (B) The mRNA level of GATA2 was correlated with the Gleason score of PCa patients in GSE21032 dataset (Gleason score≤7, n=117; Gleason score>7, n=22). (C) Kaplan-Meier plot of DFS in PCa patients from GSE21032 dataset stratified by mRNA expression level of GATA2. (D) ROC curve analysis on GATA2 in PCa patients from GSE21032 dataset. ***, p<0.001; ns, p≥0.05.

**Additional file 7: Table S3.** The antibody list.

**Additional file 8: Table S4.** The list of reagents and kits.

## Data Availability

The datasets presented in this study can be found in online repositories. The names of the repositories and accession numbers can be found in the article/Supplementary Material.

## Abbreviations

GATA2: GATA binding protein 2
PCa: Prostate cancer
TCGA: The Cancer Genome Atlas
IHC: Immunohistochemistry
SLeX: Sialyl Lewis X
PSA: Prostate-specific antigen
ST6GALNAC: ST6 N-Acetylgalactosaminide Alpha-2,6-Sialyltransferase
sTN: Sialyl-Tn
DFS: Disease-free survival
HR: Hazard ratio
CI: Confidence interval
ROC: Receiver-operating characteristic
AUC: Area Under ROC Curve
IACUC: Institutional Animal Care and Use Committees
BPH: Benign prostatic hyperplasia
GSEA: Gene set enrichment analysis
GO: Gene Ontology
KEGG: Kyoto Encyclopedia of Genes and Genomes
BP: Biological process
CC: Cellular components
MF: Molecular function
PPI: Protein–protein interaction
SRB: Sulforhodamine B
PIN: Prostatic intraepithelial neoplasia
PNI: Perineural invasion
EMT: Epithelial-to-mesenchymal transition
AR: Androgen receptor
CRPC: Castration-resistant prostate cancer
BMP6: Bone Morphogenetic Protein 6
ChIP: Chromatin immunoprecipitation
