# Supplementary figures and images for: Integrative analysis of the ST6GALNAC family identifies GATA2-upregulated ST6GALNAC5 as an adverse prognostic biomarker promoting prostate cancer cell invasion
Source: Cancer Cell Int. 2023 Jul 19;23:141. doi: 10.1186/s12935-023-02983-x (PMC10355028; doi:10.1186/s12935-023-02983-x)

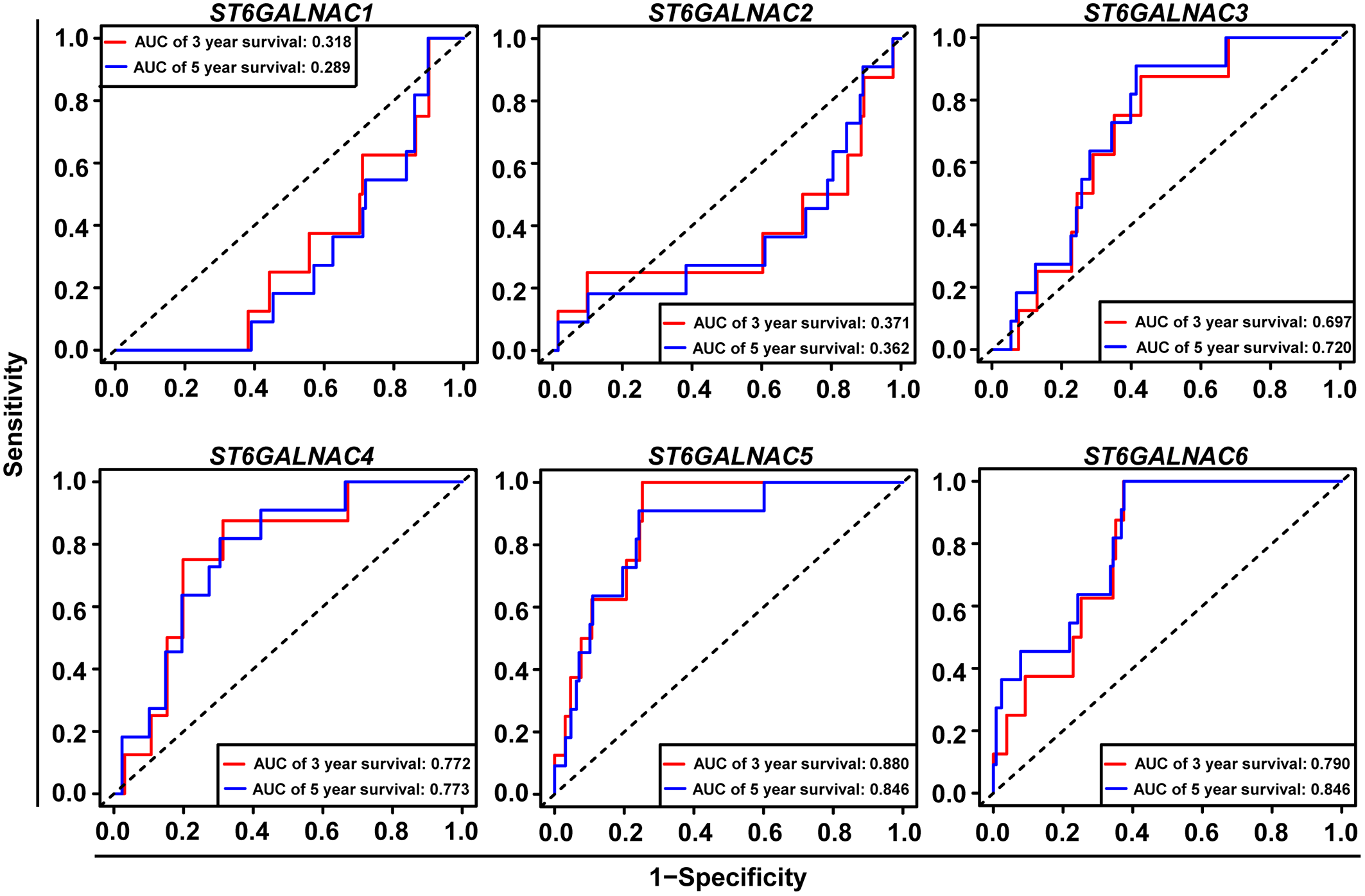

Supplement: Supplementary file 3 — Additional file 3: Figure S1. ROC curve analysis on six ST6GALNAC family members in PCa patients from GSE21032 dataset. [file 12935_2023_2983_MOESM3_ESM.tif]

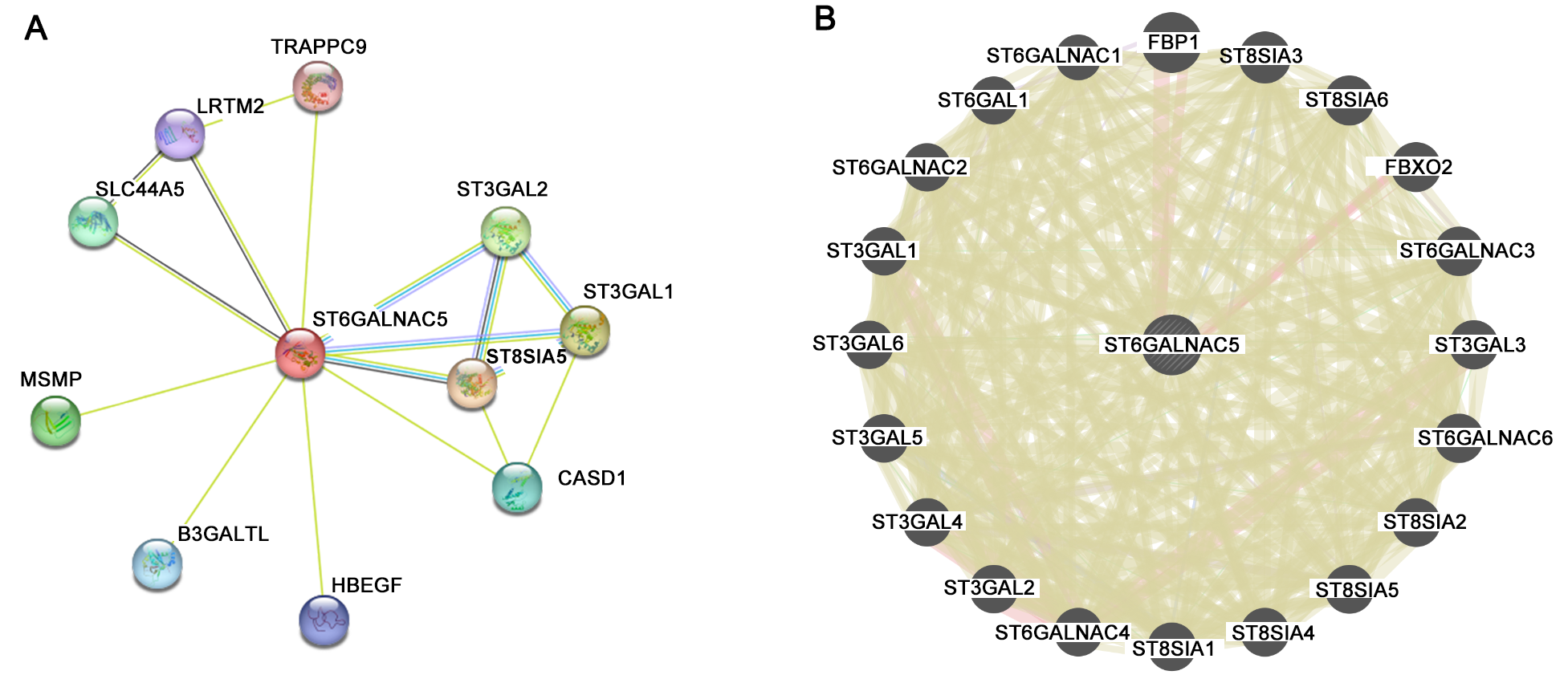

Supplement: Supplementary file 4 — Additional file 4: Figure S2. The PPI analyses of ST6GALNAC5 in PCa samples. PPI network of ST6GALNAC5 was constructed by STRING (A) and GeneMANIA (B) programs. [file 12935_2023_2983_MOESM4_ESM.tif]

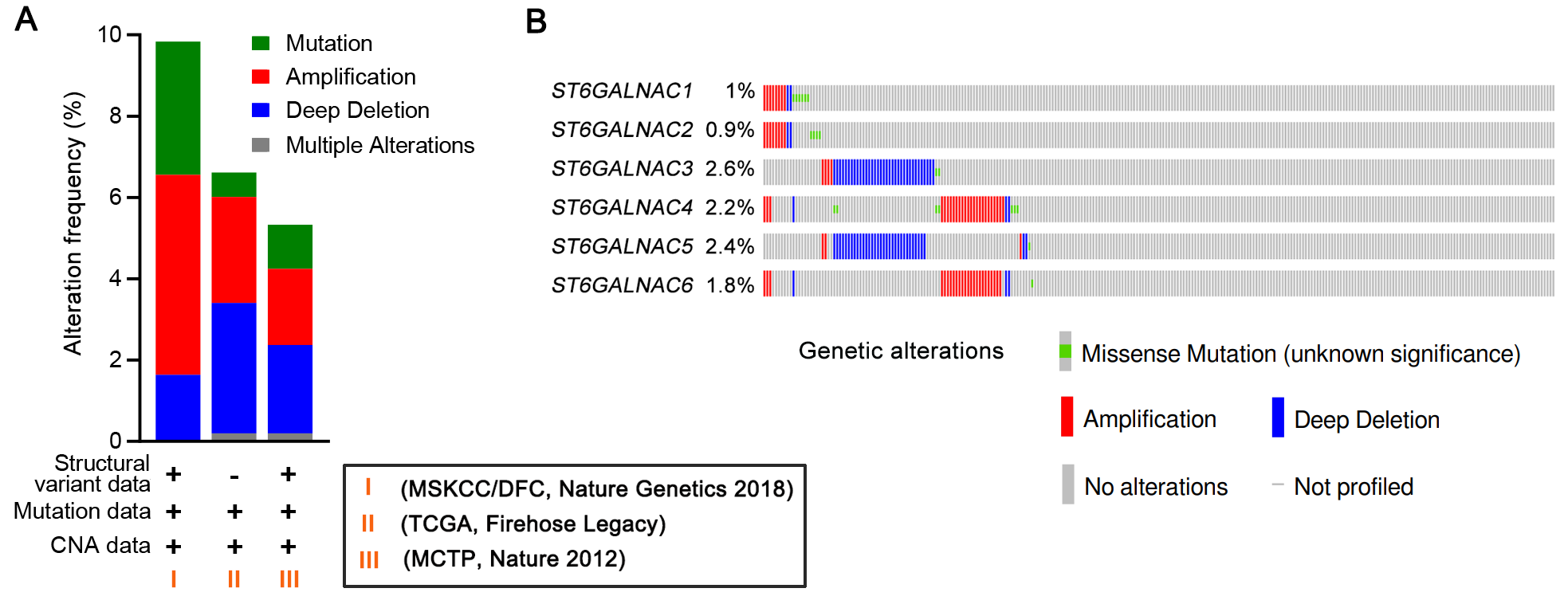

Supplement: Supplementary file 5 — Additional file 5: Figure S3. The genetic alterations of ST6GALNAC family members in PCa samples were analyzed in plot (A) and in individual cases (B) from the cBioPortal online database. [file 12935_2023_2983_MOESM5_ESM.tif]

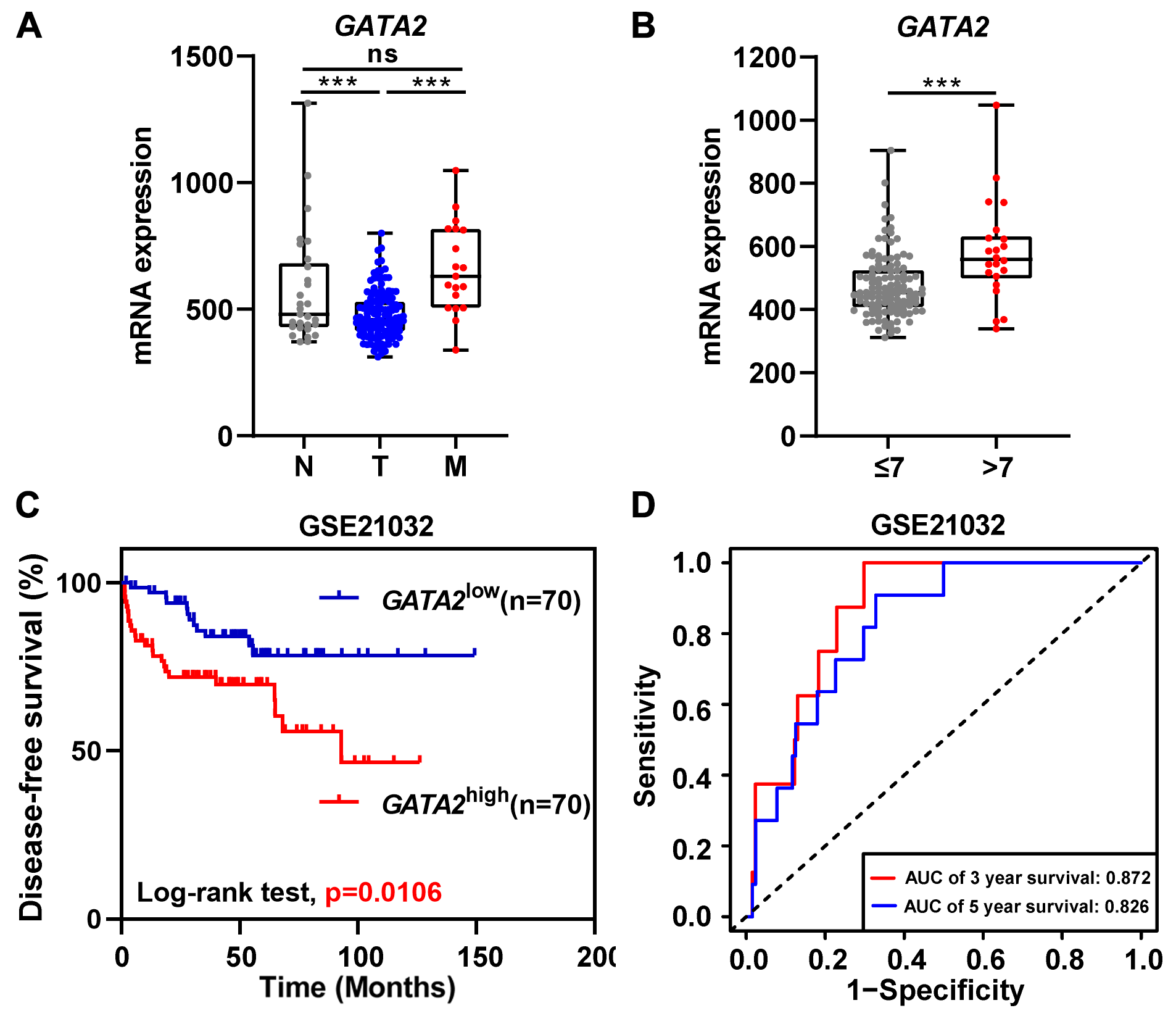

Supplement: Supplementary file 6 — Additional file 6: Figure S4. The association analysis on GATA2 mRNA expression with clinicopathological parameters of PCa patients in GSE21032 dataset. (A) The mRNA level of GATA2 in normal prostate (N, n=29), primary PCa (T, n=131) and metastatic PCa (M, n=19) in GSE21032 dataset. (B) The mRNA level of GATA2 was correlated with the Gleason score of PCa patients in GSE21032 dataset (Gleason score≤7, n=117; Gleason score>7, n=22). (C) Kaplan-Meier plot of DFS in PCa patients from GSE21032 dataset stratified by mRNA expression level of GATA2. (D) ROC curve analysis on GATA2 in PCa patients from GSE21032 dataset. ***, p<0.001; ns, p≥0.05. [file 12935_2023_2983_MOESM6_ESM.tif]
